# The Role of the Microbiome in Liver Cancer

**DOI:** 10.3390/cancers13102330

**Published:** 2021-05-12

**Authors:** Mar Moreno-Gonzalez, Naiara Beraza

**Affiliations:** 1 Gut Microbes and Health Institute Strategic Programme, Quadram Institute Bioscience, Norwich Research Park, Norwich NR4 7UQ, UK; mar.moreno-gonzalez@quadram.ac.uk; 2 Food Innovation and Health Institute Strategic Programme, Quadram Institute Bioscience, Norwich Research Park, Norwich NR4 7UQ, UK

**Keywords:** hepatocellular carcinoma, microbiota, microbiome, gut–liver axis, chronic liver disease, cirrhosis

## Abstract

**Simple Summary:**

Hepatocellular carcinoma (HCC) is the most common primary liver cancer and the fourth most common cause of cancer-related deaths worldwide; its incidence and mortality rate continue to increase. HCC has a poor prognosis and the curative options are very limited. The liver is closely connected (anatomically and functionally) to the gastrointestinal tract, which represents the largest reservoir of microbes in our body. Increasing evidence implicates communication between the liver and the intestine (and its microbiome) in the progression of chronic liver disease to liver cancer. In this review, we summarise current knowledge on the role of the gut–liver axis in contributing to the progression of HCC, with a focus on the impact of the intestinal microbiome in this process. We also review the potential of therapeutic strategies based on modulation of the microbiome for the prevention of HCC progression.

**Abstract:**

Hepatocellular carcinoma (HCC) is the most common malignancy occuring in the context of chronic liver disease and is one of the main causes of cancer-derived death worldwide. The lack of effective treatments, together with the poor prognosis, underlines the urge to develop novel and multidisciplinary therapeutics. An increasing body of evidence shows that HCC associates with changes in intestinal microbiota abundance and composition as well as with impaired barrier function, leading to the release of bacteria and their metabolites to the liver. These factors trigger a cascade of inflammatory responses contributing to liver cirrhosis and constituting an ideal environment for the progression of HCC. Interestingly, the use of bacteriotherapy in human and preclinical studies of chronic liver disease and HCC has been shown to successfully modify the microbiota composition, reducing overall inflammation and fibrosis. In this review, we explore the existing knowledge on the characterisation of the intestinal microbial composition in humans and experimental murine chronic liver disease and HCC, as well as the use of antibiotics and bacteriotherapy as therapeutic options.

## 1. Introduction

Hepatocellular carcinoma (HCC) is the most common primary liver cancer and the fourth most common cause of cancer-related deaths worldwide [1,2]. Unlike other malignancies, its incidence and associated mortality rate continue to increase [3]. In 80% of cases, HCC develops after cirrhosis has already been established, and one in three patients with compensated cirrhosis will develop HCC [4]. HCC is the leading cause of mortality in cirrhotic patients. Generally, the risk factors for cirrhosis include: alcoholic and nonalcoholic steatohepatitis, viral infection (hepatitis B or hepatitis C) and exposure to dietary toxins (e.g., aflatoxin B [1]). More specifically, due to an increase in the obesity-associated metabolic syndrome of epidemic proportions, NAFLD is becoming a leading cause of HCC in western countries, particularly in elder patients with comorbidities.

HCC has a poor prognosis, which is related to its late detection and diagnosis. HCC is a heterogeneous and highly complex disease for which treatments are aimed at improving survival rates. Early-stage curative options include liver transplantation and resection and ablation, while transarterial chemoembolization is effective at intermediate stages. In the case of advanced HCC, treatment options are limited but include first line drugs such as sorafenib and lenvanitib and second line drugs such as regorafenib (amongst many others) with more limited success [1]. Most recently, atezolizumab plus bevacizumab has shown promising effects in prolonging progression-free survival in patients with unresectable tumours [5].

Inflammation is a hallmark of the progression of chronic liver disease into cirrhosis/HCC; 85% of cancer cases occur in chronically inflamed livers. Hepatocellular death triggers necroinflammation, which promotes a compensatory regenerative response in the liver. When the injury–inflammation cycle is sustained, it activates the fibrotic response (surrounding regenerative nodules), leading to cirrhosis and loss of function [6,7]. The precise mechanisms underpinning the progression from chronic (inflammatory) liver disease to cirrhosis/HCC remain largely undefined, though it is increasingly recognised that the gut–microbiome–liver axis plays an important role.

In this review, we summarise the contribution of the gut–liver axis to the progression from chronic liver disease into cirrhosis/HCC. We focus on the role of the intestinal microbiome, in particular how the microbiome changes during the progression of chronic liver disease to HCC and the potential therapeutic effects of treatment strategies that target the microbiome.

## 2. Gut–Liver Axis

The liver and the intestine are anatomically and functionally connected in a bidirectional relationship. The liver receives the majority of its blood supply from the gastrointestinal (GI) tract via the portal circulation, which is enriched in essential nutrients that support the metabolic function of the liver. In turn, the intestine receives different metabolites from the liver, including bile acids that are synthesised in the liver and transported into the intestine, where they regulate intestinal microbiome composition due to their antimicrobial properties [8].

The intestinal barrier is a multilayered defence system against external pathogens. This barrier includes the intestinal epithelium, a mucus layer and the mucosal-associated immune system. Together, they maintain a delicate symbiotic equilibrium between residing microbiota and the host—mainly by preventing their direct contact with each other [9]. Ultimately, the gut–vascular barrier avoids the dissemination of intestinal microbes into the systemic circulation. Despite this, the enterohepatic circulation can contain food antigens and bacterial components that have escaped the intestinal mucosal/vascular barrier, and reached the liver. Thus, the liver is the body’s second firewall, and it relies on its strong immune system to detect and eliminate gut-derived substances (including bacteria), thereby protecting the host and preserving whole-body homeostasis [10].

In the liver, the innate immune system is the first line of defence against infection and damage, and it must balance ‘recognition of self’ with ‘responses to challenge’. Macrophages play a key role in defence via the phagocytosis of cell debris (i.e., danger-associated molecular products (DAMPS)) and microbial/pathogen-derived products (MAMPS/PAMPS). Macrophages detect these products via pathogen recognition receptors (PPRs), including Toll-like receptors (TLRs); the activation of TLRs promotes inflammation [11]. The intestinal immune system is also characterised by a tolerogenic function aimed at avoiding hyper activation of immune cells in response to commensal microbes colonising the GI tract during homeostasis, as well as its tolerance to food antigens [12].

The GI tract is home to the greatest concentration of microbes in the body [13] where they have a mutualist relationship with the host [14]. The term microbiome refers to the collective genome of the microbes colonising the gut while the microbiota represents the complex ecosystem comprised of diverse bacteria, viruses, archaea and fungi that coexist and interact with the host [15]. The intestinal microbiota is metabolically and immunologically active and benefits host health. This includes the extraction of nutrients from indigestible fibres, the preservation of tissue homeostasis and protection from pathogens. This relationship is reciprocal; the microbiota can be shaped by diet, lifestyle, age, antibiotics and disease progression [16].

The liver shapes gut microbiome composition via the synthesis and transport of bile acids (which have antimicrobial properties) into the intestine, where the generation of IgA antibodies ensues. Reciprocally, the intestinal microbiome metabolises liver-derived bile acids into secondary products that are recycled back into the liver [17]. The intestinal microbiome also generates a variety of metabolites after food fermentation, including trimethylamine (TMA), vitamins and short-chain fatty acids (SCFA) that influence host immunity, barrier function and overall liver function [18].

This bidirectional relationship is greatly disturbed during chronic disease where small intestine bacterial overgrowth (SIBO) and changes in microbiome composition (i.e., dysbiosis) [19,20] associated with increased intestinal permeability (the ‘leaky gut’) [20,21] allow endotoxins into the systemic circulation [22,23,24], the presence of which correlates with disease progression [25]. However, our understanding of the mechanisms of these relationships are still limited and only recently accepted.

In the next section we will summarise more specifically how the microbiome composition changes during chronic liver disease, cirrhosis and HCC and the current knowledge on how bacteria impact on disease progression.

## 3. The Intestinal Microbiome and Progression to HCC

### 3.1. Differences in Microbiome Composition and Progression to HCC

Bacteria are the most abundant micro-organisms in the GI tract of humans; Firmicutes and Bacteroidetes are the predominant phyla and Actinobacteria, Proteobacteria and Verrucomicrobia are also present [26,27]. The development of culture-independent technologies (e.g., the targeted sequencing of the 16S ribosomal RNA (rRNA) gene) and novel computational techniques has enabled the characterisation of microbiome composition at a higher taxonomic resolution than previously possible, and these have been used to define microbiome composition during the progression of liver disease. Most recently, untargeted whole-DNA metagenomic sequencing has enabled increased taxonomic resolution to the species level and has also, critically, enabled attribution to functional differences in the microbiome during disease progression [26,28].

Associations between particular microbiome compositions and the development of HCC (generally from cirrhosis) are well supported in numerous studies of patients with HCC arising from different aetiologies, and we describe these in the sections below, with particular reference to alcoholic liver disease (ALD), nonalcoholic fatty liver disease/nonalcoholic steatohepatitis (NAFLD/NASH) and viral hepatitis (summarised in Table 1). Despite this, most available studies in patients describe differences in the microbiome at different stages of disease but not causality. While these studies are extremely relevant, studies demonstrating causality and the role of the microbiome as an active driver of liver disease progression are still scarce.

#### 3.1.1. Microbiome and ALD

Alcohol is toxic to epithelial cells and contributes to the disruption of the intestinal barrier [60] and an increase in permeability [24,61,62] that, together with SIBO [21], promote endotoxemia in ALD patients. Several studies have described significant changes in the composition of the microbiome in ALD patients (summarised next and in Table 1). These changes in composition, as well as in the SIBO observed, may result from several factors, including an altered diet (generally low in proteins), alcohol itself and its metabolite acetaldehyde, altered bile acid flow and metabolism, reduced gastrointestinal motility and mucosal immune defence in the gut of ALD patients [63].

In colonic biopsies from ALD patients, the abundance of Enterobacteriaceae and Proteobacteria was higher than that in biopsies from healthy patients and the abundances of Bacteroidetes and Firmicutes were lower [29]; there was a skew towards a greater presence of potentially pathogenic bacteria such as Enterobacteriaceae, Veillonelaceae and Streptocacceae, with a lower presence of Lachnospiraceae in ALD patients [50]. The microbiome composition of patients varied in relation to the severity of disease; levels of *Bifidobacteria* and *Streptococci* were highest in severe alcohol hepatitis cases, while Enterobacteria was enriched in all cases [30].

Mechanistically, in murine models of ALD, lower abundances of Firmicutes and higher abundances of Verrucomicrobia and Bacteroidetes were observed when compared with those in healthy mice [64]; the dysbiotic microbiome contributed to the progression of liver disease via the promotion of intestinal inflammation and the resulting reduction in tight junction protein expression, which was similar to our findings in murine models of cholestasis [65]. Depletion of antimicrobial molecules was also demonstrated in murine models of ALD [64,66].

#### 3.1.2. Microbiome and NAFLD/NASH

As with ALD, SIBO and increased intestinal permeability are key features of NAFLD/NASH in patients [67,68,69,70]. Changes in the microbiome composition have been described as well. Thus, microbiome composition is different in NAFLD [32,33] and NASH [34,35] in adults, and these changes correlate with disease severity. Additionally, in children, NAFLD and NASH [38,39] were associated with a different microbiome composition that correlated with the progression of the disease and was characterised by an increased ethanol production [38].

In adults, the abundance of *Escherichia coli* is greatest in NASH individuals compared to that in obese individuals [35]. Furthermore, the abundance of Proteobacteria was greater in NAFLD patients with fibrosis [32], and the abundance of *Ruminococcus* was higher in NASH patients with advanced fibrosis compared with that in nonfibrotics [34]. In a recent large study, a NAFLD–cirrhosis microbial signature was identified; NAFLD was characterised by an increased abundance of *Megasphaera* and a reduced abundance of *Faecalibacterium prausnitzzi*, denoting a predominance of Gram-negative bacteria during cirrhosis [36]. Another study focusing on an Italian cohort of NAFLD patients showed an enrichment of Enterobacteriaceae and the genus *Streptococcus* during cirrhosis compared with their levels in healthy controls [37]. Importantly, recent molecular phenomics and metagenomics characterisation of the microbiome of obese females has demonstrated the increase in LPS-producing proteobacteria [71], which has been shown to be consistently enriched in NAFLD/NASH [72]. These studies have also demonstrated the contribution of microbial metabolism (i.e. increased utilisation of branched chain amino acids and the degradation of aromatic amino acids) in contributing to liver steatosis and the key role of phenylacetic acid in promoting liver steatosis in mice [71].

Importantly, it was demonstrated that the likelihood of developing fibrosis in the context of NAFLD could be transmitted from patients with cirrhosis to first-degree relatives, who were at increased risk of developing advanced fibrosis [73]. More detailed information about specific signature microbiota during NAFLD can be found in [74] and in Table 1.

#### 3.1.3. Microbiome and Viral Hepatitis

Patients with hepatitis B virus (HBV) had a higher abundance of the genera *Veillonella*, *Prevotella* and *Clostridium (senso stricto*), as well as Lachnospiraceae, and a lower abundance of the genera *Alistipes* and *Bacteroides* compared with those of healthy controls [40,41,42]. For HBV patients with decompensated cirrhosis, *F.prausnizzi* and *Enterococcus fecalis* were predominant while the abundance of *Bifidobacterium* species and lactic acid bacteria decreased (Table 1) [43].

Hepatitis C Virus patients (HCV), both with and without evidence of cirrhosis, showed high abundances of the genera *Bacteroides*, *Staphylococcus*, *Veillonella*, *Prevotella* and *Streptococcus*, and low abundances of the genera *Clostridium* and *Ruminococcus* (among others) compared with those of healthy controls [44,45,46] (Table 1).

#### 3.1.4. Microbiome and Cirrhosis

In cirrhotic patients, SIBO [21,75] and the disruption of intestinal barrier function [76] allow for bacterial translocation, which leads to systemic inflammation [77]. Endotoxemia is associated with ascites and spontaneous bacterial peritonitis as well as portal hypertension, varices and hepatic encephalopathy (HE); all of which are common complications in decompensated cirrhotic patients [6,7,25,78,79].

Several studies have described profound differences in the microbiome composition of cirrhotic patients compared with those of controls [47,49,50,51,52,56]. Cirrhotic patients showed a dominance of *Streptococcus*, *Veillonella* and *Enterococcus* genera and low abundances of Lachnospiraceae and Ruminococcaceae [47,50,52,53,54]. Some of these differences were positively correlated with blood ammonia levels [46,80] and other complications such as HE [47,48,52] and ascitis [55], but appeared to be independent of the aetiology [56]. Differences in the microbiome composition of cirrhotic patients compared with healthy controls was associated with poor cognition, inflammation and disease severity, which supports the hypothesis that the microbiome is influential in the gut–brain–liver axis [48,53] (all summarised in Table 1)

Functional characterisation of the cirrhosis-associated microbiome using metagenomic analyses highlighted that ammonia and endotoxin production, alongside membrane transport, were the predominant functions of the cirrhotic microbiome [52].

Increasing evidence supports the relevance of the oral microbiome during cirrhosis. In cirrhotic patients with associated HE, the salivary microbiota was profoundly different to that of controls and was associated with systemic inflammation [54]. Furthermore, stool samples from over 50% of cirrhotic patients were enriched with members of the oral microbiome, including the genera *Veillonela*, *Streptococcus* and *Prevotella*. These findings support the idea that oral commensals invade the intestinal tract during cirrhosis, which is likely to result in profound changes in the composition of the bile acid pool and the subsequent colonisation and growth of opportunistic (oral) species in the intestine [31,52]

#### 3.1.5. Microbiome and HCC

Microbiome analysis using 16S rRNA sequencing showed that HCC patients from the NASH aetiology had a distinct composition when compared to NASH-cirrhotic patients. The HCC microbiome was associated with lower diversity, with significantly higher abundances of bacterial genera related to anti-inflammatory functions (e.g., *Bifidobacterium* and *Blautia*); and significantly higher abundances of the genera *Enterococcus*, *Ruminococcus*, *Bacteroides*, *Phascolarctobacterium* and *Oscillospira* [37].

In addition, the microbiome composition of patients with HCC progressing from viral hepatitis was different to that of nonrelated viral hepatitis–HCC patients and to that of controls; the viral hepatitis–HCC patients had a higher species richness and were enriched in *Prevotella* compared to both nonrelated viral hepatitis–HCC patients and healthy controls. Nonrelated viral hepatitis–HCC patients were dominated by proinflammatory bacterial genera such as *Enterococcus* and *Escherichia*-*Shigella* and had lower abundances of the genera *Ruminococcus* and *Faecalibacterium* compared with viral hepatitis–HCC patients [57]. The abundance of *E coli* was different in patients with cirrhosis compared with patients with both cirrhosis and HCC [58].

Most recently, the detection of the differences in faecal microbiome composition during early HCC compared with healthy controls has been proposed as a noninvasive biomarker [59].

### 3.2. Impact of Bacterial Components in HCC Development

Our mechanistic understanding of the role the microbiome plays in the development of HCC has been generated mainly in preclinical models using rodents [81,82,83,84]; these studies have shown how the activation of the TLR4 signalling pathway in response to LPS, as well as the direct toxic effects of secondary bile acids (which are microbial metabolites) in the liver, promote HCC development.

In a seminal study, Seki et al. demonstrated the key role of LPS–TLR4 signalling in the mediation of fibrosis progression in mice [85]. Further studies used antibiotics in rodent models as a tool for understanding disease progression in the absence the gut microbiota; in this way, Dapito and colleagues were able to demonstrate that the depletion of the microbiota protected against fibrosis and HCC development in mice. Furthermore, germ-free mice had fewer tumours than did animals with a conventional microbiome, overall supporting the role of the LPS-TLR4 axis during tumorigenesis [81]. Likewise, neomycin protected against HCC development after diethylnitrosamine/carbon tetrachloride (DEN/CCl_4_) [83].

Secondary bile acids have also shown to regulate immune function and HCC development. For example, deoxycholic acid (DCA) contributed to liver inflammation by promoting the senescence-associated secretory phenotype (SASP), overall contributing to obesity-related HCC development [82]. A further study showed that DCA induced nonalcoholic steatohepatitis (NASH)-associated HCC by activating mTOR [86]. Changes in the bile acid pool after antibiotic treatment (resulting in a reduction in secondary bile acid and an increase in primary bile acid) led to increased antitumour immunity (NKT cell activation after primary bile acid production; TbMCA) [87]. Antibiotics also significantly reduced DCA levels and, thus, liver inflammation and NASH-related HCC development [82].

The demonstrated associations between the gut–microbiome–liver axis and progression of chronic liver disease to cirrhosis and further into HCC suggests that management strategies targeting the microbiome have therapeutic potential.

## 4. HCC and Bacteriotherapy

Curative options for advanced HCC are very limited, and driving the need to develop new therapeutics. To improve their efficacy, these therapeutics should focus on preventing the progression from chronic liver disease to cirrhosis and from compensated to decompensated cirrhosis. Bacteriotherapy could restore microbiome composition, reduce intestinal permeability (and thus reduce endotoxemia) and attenuate the chronic inflammatory environment in the liver; in this way, there is the potential that progression of disease and tumour development could be delayed or halted. Ideally, these therapeutic approaches would be more effective if they targeted earlier stages of disease and aimed to reduce chronic inflammation and cirrhosis, rather than directly reduce tumour mass.

While the association between microbiome composition and the development of HCC is well studied and accepted, available evidence on the active role of differences in the microbiome (c.f., healthy individuals) as a driver of HCC development is mostly restricted to preclinical studies in rodent models (reviewed in this section and in Table 2).

### 4.1. Antibiotics

Antibiotic use has had beneficial outcomes in ALD treatment via the restoration of the barrier function in rodents [88,89]. Antibiotics also attenuated NAFLD by modulating intestinal-regulated bile acid metabolism in mice [90]. Rifaximin, an unabsorbed antibiotic, also reduced the proinflammatory phenotype of immune cells (with an increased Th1 pattern) in the intestine of cirrhotic rats [91].

The efficacy of antibiotics in the treatment of cirrhosis expands their use in the preclinical setting. Rifaximin showed success in palliative treatment of patients for complications associated with cirrhosis; it reduced encephalopathy and variceal bleeding, and increased the survival of cirrhotic patients [92,93]. Furthermore, rifaximin and norfloxacin reduced the incidence of spontaneous bacterial peritonitis in cirrhotic patients [19,94,95].

Available evidence of the efficacy of antibiotics during development of HCC is restricted to animal studies, which show beneficial effects of antibiotic treatment in reducing tumour size after DEN treatment [83]. Antibiotics also reduced HCC progression in a DEN/CCL4 experimental mouse model [81] and in an obesity-related HCC model [82].

Although promising, these results may not be fully applicable in a clinical setting, as potential deleterious effects of long-term antibiotic use must be taken into consideration. These include negative effects of the capacity of the gut microbiome to regulate host intestinal function [114] and the toxic effects of antibiotics on kidney function amongst other side effects. These could be circumvented using ‘safe’ antibiotics like norfloxacin. Nonetheless, long-term treatments with antibiotics can lead to antimicrobial resistance (AMR) [115,116]; multidrug-resistant infections, particularly in decompensated cirrhotic patients, are a key consideration [117,118].

### 4.2. Probiotics

Probiotics are defined as “live microorganisms that, when administered in adequate amounts, confer a health benefit on the host” [119]. The use of probiotics during chronic liver disease, cirrhosis and (preclinical) HCC is aimed at restoring gut microbiome composition and improving barrier function, thus attenuating gut–liver inflammation.

During ALD, the administration of *Lactobacillus rhamnosus* GG reduced endotoxemia, liver inflammation and injury in mice [96,97]. In alcoholic patients, administration of *Bifidobacterium bifidum* and *Lactobacillus plantarum* attenuated liver injury markers and were associated with the restoration of intestinal microbiota composition compared with patients following the standard therapy alone [103].

In the context of NASH, *Lactobacillus casei* has been shown to protect against steatosis in rats fed on a high fat diet [98,99]. The benefits of probiotics in attenuating NAFLD/NASH were further observed in several human studies where different probiotics (including *Lactobacillus* and *Bifidobacterium* species) and synbiotics attenuated liver injury and steatosis in patients (reviewed in [120]). The probiotic VSL#3 efficiently reduced NASH in obese children [101].

There is also evidence of the efficacy of different probiotics during cirrhosis. Thus, in alcoholic- and NASH-cirrhotic patients, the commercially-available probiotic VSL#3 (containing four *Lactobacillus*, three *Bifidobacterium* and one *Streptococcus thermophilus* subsp salivarius) improved liver function, reduced systemic inflammation [102] and reduced the hospitalisation time of cirrhotic patients with associated HE [105]. Cirrhotic patients receiving nonpathogenic *E coli* Nissle had reduced endotoxemia and improved liver function [106], and treatment with *L.casei* Shirota restored neutrophil dysfunction associated with alcoholic cirrhotic patients, improving their phagocytic capacity [104].

Available studies on the use of probiotics as therapeutics for HCC have all been done on preclinical models. *L.rhamnosus* GG, *E coli* Nissle and heat-inactivated-VSL#3 limited the growth of liver tumours in mice [100]. The underlying mechanisms involved a reduction in the abundance of IL17A-producing segmented filamentous bacteria. Administration of VSL#3 also significantly reduced tumour number and size after DEN treatment in rats [84] via mechanisms including the restoration of intestinal barrier function and a reduction in inflammation.

Overall, though very promising, there remains a need for new well-designed, larger-scale and robustly powered clinical studies to support the use of probiotics to treat chronic liver disease, cirrhosis and progression to HCC.

### 4.3. Faecal Microbiota Transplantation (FMT)

FMT is highly effective for treatment of *Clostridium difficile* infections [121,122], while its efficacy in the treatment of chronic liver disease is still under current investigation. In mice, FMT alleviated high fat diet-induced steatosis [109] and prevented alcohol-induced steatosis and liver inflammation in mice [108]. FMT was also beneficial for prevention of HE in rats treated with CCl_4_ [107].

In patients, FMT has been shown to: improve insulin sensitivity in patients with metabolic syndrome [110], improve cognitive function in patients with cirrhosis and HE [111]; and restore antibiotic-associated disruption in microbial diversity and function in patients with advanced cirrhosis [112]. Recently, the PROFIT study demonstrated the safety of FMT in advanced and stable cirrhosis where it reduced ammonia plasma levels in patients that had received no previous antibiotic treatment [113].

The efficacy of FMT, with or without associated antibiotic treatment, should be further evaluated alongside establishing the highest standards for donor selection and sample processing before FMT.

### 4.4. Microbiome and Cancer Immunotherapies

There is increasing evidence for microbiome involvement in the beneficial regulation of the efficacy of therapeutics that stimulate anticancer responses [123,124,125].

For example, the efficacy of cyclophosphamide was reduced in tumour-bearing mice treated with antibiotics and in germ free conditions, supporting the key role of the microbiome in regulating host-immune responses that control cancer growth [125].

Likewise, several studies have demonstrated that the microbiome can affect the efficacy of immunotherapies based on immune checkpoint inhibitors (using PD-L1 antibodies and CTLA-4) in different cancers [126,127,128,129,130]. In a study of melanoma patients, there was a higher abundance of Ruminococcaceae and Bifidobacteriaceae (e.g., *B.longum*) in the intestinal microbiome of patients responding to PD-L1 therapy compared with patients not responding to the therapy [126,127]. Preclinical studies in mice demonstrated the beneficial effect of supplementation with *Bifidobacterium* species on the anti-tumour efficacy of anti-PDL1 and anti-CTL4 [129,130], while antibiotics negatively affected the efficacy of PD-1 and CTL-4 antibody therapy in mice and patients [128].

Overall, these important findings point to the potential of modulating the efficacy of immune checkpoint therapies via microbiome-based strategies. Moreover, the effects of long-term antibiotic treatments (reviewed in Section 4.1) alongside studies demonstrating the role of the commensal microbiome in modulating the effects of anticancer immune therapies (reviewed in Section 4.4), support the use of alternative therapeutic strategies to modulate the microbiome including the use of probiotics and FMT (reviewed in Section 4.2 and Section 4.3).

## 5. Conclusions

The contribution of the gut microbiome to chronic liver disease and the development of HCC has been acknowledged in the last years with, massive advances being made in characterising the microbiome associated with different liver disease aetiologies and severities. In addition, mechanistic work in rodent models has established the contribution of the intestinal microbiome to the progression of liver disease. However, these studies have focused on the role of bacterial endotoxin (LPS), while a thorough analysis of the contribution of specific bacteria (or defined communities) to chronic liver disease remains undefined. Thus, we still lack of a detailed understanding on the individual effects of the different microbes found to be modulated during human chronic disease and their specific contributions to the progression of the disease. Likewise, our knowledge of the mechanisms mediating both the detrimental and the beneficial effects of different bacteria on liver function and disease progression remain scarce. These may include the capacity of specific bacteria to regulate tight junctions and, thus, intestinal permeability [65], controlling inflammation [66], or via the direct effects of bacteria-derived metabolites (e.g., phenylacetic acid) on the liver, as it was observed in obese steatotic patients [71].

Greater understanding about bacterial function, its impact on the host, its contribution to the loss of barrier function and the gut–liver immune system will lay the foundations for novel therapeutic approaches to the treatment of chronic liver disease that will attenuate progression to cirrhosis and HCC. This will also enable us to design specific therapeutics for different stages of disease, as each may require different strategies, particularly in combination with other therapies (such as immune checkpoints). Some of these approaches may involve the modulation of microbiome composition and/or its function directly or indirectly (as reviewed in [131]) using dietary approaches, probiotics, prebiotics, symbiotics or bacteriophages [132].

For this, improved functional analysis of the microbiome and more mechanistic studies are paramount to move from pre-clinical models to the clinical setting. These will refine our understanding not only of which microorganisms are present but also of what they are doing and how functionally relevant the observed differences in microbiome composition are. It will also enable us to know whether these differences in microbiome can be used as biomarkers of disease susceptibility and progression. Ultimately, more robust and sufficiently powered human studies are crucial to evaluate the efficacy of live bacteria therapies for the treatment of liver disease progression and translate them into clinical practice.

## Figures and Tables

**Table 1:** Differences in microbiota composition during chronic liver disease and hepatocellular carcinoma (HCC).

Microbiota Composition in ALD Patients Compared with Healthy Controls											
Patients	Sample	Method	Class Level		Family Level		General Level		Specie Level		Reference
			Increased	Decreased	Increased	Decreased	Increased	Decreased	Increased	Decreased	
ALD	Colonic Biopsy	16s rRNA LH-PCR and multitag pyrosequencing (MTPS)	↑ Bacili, Gammaproteobacteria	↓ Bacteroidetes, Clostridia		↓ Bacteroidaceae					[29]
ALD + alcoholic hepatitis (AH)	Stool	16S rRNA pyrosequencing			↑ Bifidobacteriaceae, Streptococcaceae, Enterobacteriaceae			↓ *Atopobium*		↓ *Clostridium leptum*	[30]
ALD	Stool	Shotgun+ Sequencing (SOLiD 5500 platform)					↑ *Streptococcus, Bifidobacterium, Lactobacillus* species, *Veillonella*	↓ *Prevotella*, *Paraprevotella*, *Alistipes*	↑ *Bifidobacterium longum*, *B.dentium*, *B.breve*, *Streptococus thermophilus*, *S.mutants*, *Lactobacillus salivarius*, *L.antri*, *L.crispatus*		[31]
**Microbiota Composition in NAFLD and NASH Patients Compared with Healthy Controls**											
**Patients**	**Sample**	**Method**	**Class Level**		**Family Level**		**General Level**		**Specie Level**		**Reference**
			**Increased**	**Decreased**	**Increased**	**Decreased**	**Increased**	**Decreased**	**Increased**	**Decreased**	
NAFLD + Fibrosis	Stool	Whole-genome shotgun sequencing of DNA							↑ *B. vulgatus*, *E. coli*	↓ *Ruminococus obeum* CAG:39, *R.obeum*, *E.rectale*	[32]
NASH	Stool	Quantitative real-time PCR							↑ *C.cocoides*		[33]
NAFLD	Stool	16S rRNA sequencing (Illumina MiSeq)			↑ Bacteroidaceae	↓ Bacteroides, Ruminococcus	↑ *Prevotella*				[34]
NASH + Fibrosis	Stool	16 S rRNA pyrosequencing			↑ Prevotellaceae, Enterobacteriaceae	↓ Bifidobacteriaceae, Lachanospiraceae, Ruminococcaceae	↑ *Prevotella*, *Peptoniphilus*, *Escherichia*	↓ *Bifidobacterium*, *Alistipes*, *Blautia*, *Coprococcus*, *Eubacterium*, *Roseburia*, *Oscillospira*, *Ruminicoccus*			[35]
NAFLD + Cirrhosis	Stool	16S rRNA sequencing (Illumina MiSeq)			↑ Enterobacteriaceae	↓ Rikenellaceae, Mogibacterium, Peptostreptococcaceae	↑ *Streptococcu*, *Gallibacterium*, *Megasphaera*	↓ *Catenibacterium*			[36]
NAFLD + Cirrhosis	Stool	16S rRNA sequencing			↑ Enterobacteriaceae, Lactobacillaceae, Pasteurellaceae, Rikenellaceae, Prevotellaceae, Bacteroidaceae, Porphyromonadaceae, Barnesillaceae, Streptococcaceae, Enterococcaceae, Veillonellaceae	↓ Verrucomicrobiaceae, Methanobacteriaceae	↑ *Lactobacillus*, *Haemophilus*, *Klebsiella*, *Prevotella*, *Parabacteroides*, *Phascolarctobacterium*, *Veillonella*, *Enterococcus*, *Pseudomonas*, *Streptococcus*, *Bacteroides*, *Atopobium*, *Dialister*, *Ruminococcus*, *Christensenella*	↓ *Akkermansia*, *Methanobrevibacter*			[37]
NAFLD + Cirrhosis + HCC	Stool	16S rRNA sequencing					↑ *Phascolarctobacterium*, *Enterococcus*, *Streptococcus*, *Gemella*, *Bilophila*	↓ *Akkermansia*, *Bifidobacterium*, *Dialister*, *Collinsella*, *Adlercreutzia*			[37]
NAFLD Obese children	Stool	16S rRNA shotgun sequencing	↑ Clostridia	↓ Alphaproteobacteria			↑ *Prevotella*				[38]
NAFLD + Steatosis	Stool	16 rRNA pyrosequencing					↑ *Bradyrhizobium*, *Anaercoccus*, *Peptoniphilus*, *Propionibacterium acnes*, *Dorea*, *Ruminococcus*	↓ *Oscillospira*			[39]
**Microbiota Composition in Viral Hepatitis Patients Compared with Healthy Controls**											
**Patients**	**Sample**	**Method**	**Class Level**		**Family Level**		**General Level**		**Specie Level**		**Reference**
			**Increased**	**Decreased**	**Increased**	**Decreased**	**Increased**	**Decreased**	**Increased**	**Decreased**	
Chronic Hepatitis B (early stage)	Stool	16S rRNA sequencing (Illumina MiSeq)			↑ Act inomycetaceae, Clostridiaceae, Lachnospiraceae, Veillonellaceae	↓ Bacteroidaceae, Coriobacteriaceae, Enterobacteriaceae, Lachnospiraceae, Porphyromonadaceae, Rikenellaceae, Ruminococcaceae	↑ *Actinomyces*, *Clostridium sensu stricto*, Unclasssified *Lachnospiraceae*, *Megamonas*	↓ *Bacteroides*, *Asccharobacter*, Unclassifiied *Coriobacteriaceae*, *Escherichis*/*Shigella*, Unclassified *Enterobacteriaceae*, Unclassified *Lachnospiraceae*, *Butyricimonas*, *Parabacteroides*, *Alistipes*, *Clostridium* IV, *Ruminococcus*, Unclassified *Ruminococcaceae*, Unclassified *Bacteria*, Unclassified *Clostridiales*			[40]
Hepatitis B + Cirrhosis	Stool	High-throughput Illumina/Solexa sequencing + Real time PCR			↑ Enterobacteriaceae, Veillonellaceae, Streptococcaceae		↑ *Veillonella*	↓ *Bacteroides*, *Clostridium*	↑ *E.coli*, *Veillonella dispar*, *V.parvula*, *Klebsiella pneumonia*, *Enterobacter cloaca*, *Shigella dysenteriae*, *S.fleneri*, *Salmonela enteric*, *Enterobacter cancerogenus*, *E.albertii*	↓ *Bacteroides cellulosilyticus*, *B.intestinalis*, *B.uniformis*, *B.ovatus*, *B.fragilis*, *B.thetaitaomicron*, *Bacteroides* sp.D1, *B.eggerthii*, *B.stercoris*, *B.vulgatus*	[41]
Chronic Hepatitis B	Stool	16S rRNA sequencing (Illumina HiSeq)					↑ *Bacteroides*, *Prevotella*, *Atopobium*, *Veillonella*, *Alistipes*				[42]
Hepatitis B + Cirrhosis	Stool	16S 16S rRNA sequencing (Illumina HiSeq)rRNA					↑ *Bacteroides*, *Akkermansia*, *Atopobium*, *Prevotella*, *Parabacteroides*				[42]
Chronic Hepatitis B	Stool	Quantitative PCR 16S rRNA						↓ *Lactobacillus*, *Pediococcus*, *Leuconostoc*, *Weisella*			[43]
Hepatitis B + Cirrhosis	Stool	Quantitative PCR 16S rRNA						↓ *Clostridium* XI, *Clostridium* XIVab			[43]
Hepatitis C + Cirrhosis	Stool	16S rRNA sequencing (Illumina MiSeq)					↑ *Prevotella*, *Faecalibacterium Acinetobacter*, *Veilonella*, *Phascolarctobacterium*	↓ *Ruminococcus*, *Clostridium*, *Parabacteroides*, *Butyricimonas*			[44]
Hepatitis C + Cirrhosis	Stool	16S rRNA sequencing (Illumina)					↑ *Streptococcus*, *Veillonela*, *Lactabacillus*, *Alloprevotella*, *Akkermansia*, *Bifidobacterium*, *Escherichia*/*Shigella*, *Haemophilus*, *Micrococcus*, *Weissella*, *Citrobacter*, *Clostrdium sensu stricto*, *Pediococcus*	↓ *Bilophila*, *Clostridium* IVV, *Clostridium* XIVb, *Mitsuokella*, *Vampirovibrio*, *Butyricimonas*, *Victivallis*			[45]
Hepatitis C (no evidence of cirrhosis)	Stool	16S rRNA sequencing (Illumina MiSeq)		↓ Clostridia	↑ Enterobacteriaceae	↓ Lachnospiraceae, Ruminococcaceae	↑ *Bacteroides*	↓ *Anaerostipes*, *Blautia*, *Fusicaatenibacter*, *Lachnospiraceae incertae sedis*, *Roseburia*, *Faecalibacterium*, *Ruminococcus*			[46]
**Microbiota Composition in Liver Cirrhosis Patients Compared with Healthy Controls**											
**Patients**	**Sample**	**Method**	**Class Level**		**Family Level**		**General Level**		**Specie Level**		**Reference**
			**Increased**	**Decreased**	**Increased**	**Decreased**	**Increased**	**Decreased**	**Increased**	**Decreased**	
Liver cirrhosis	Stool	16S rRNA Multitag pyrosequencin (MTPS)			↑ Enterococcaeae, Staphylococcaceae, Enterobacteriaceae	↓ Clostridiales XIV, Ruminococcacae, Lachnospiraceae					[47]
Liver cirrhosis	Stool and colonic mucosa	16S rRNA+multitag pyrosequencing (MTPS)+ Real-time PCR					↑ *Enterococcus*, *Proteus*, *Clostridium*, *Burkholderia*	↓ *Dorea*, *Subdoligranulum*, *Incertae Sedis* other			[48]
Liver cirrhosis	Stool	16S rRNA Multitag pyrosequencin (MTPS)			↑ Enterobacteriaceae, Fusobacteriaceae, Alcaligenaceae, Lactobacillaceae, Leuconostocaceae	↓ Lachnospiraceae, Ruminococceae, Clostridium Incertae sedis XIV					[49]
Liver cirrhosis	Stool	16S rRNA pyrosequencing + real time PCR	↑ Gammaproteobacteria, Bacilli	↓ Bacteroidetes	↑ Enterobacteriaceae, Pasteurellaceae, Streptococcaceae, Fusobacteriaceae, Veillonellaceae	↓ Lachnospiraceae, Bacteroidaceae	↑ *Enterococcus*, *Clostridium* XI				[50]
Liver cirrhosis	Stool	16S rRNA real-time PCR			↑ Enterobacteriaceae, Enterococcus			↓ *Bifidobacterium* (no statistical significance)			[51]
Liver cirrhosis	Stool	Illumina HiSeq sequencing				↓ Lachnospiraceae, Ruminococcaceae	↑ *Veillonella*, *Streptococcus*, *Clostridium*, *Prevotella*, *Campylobacter*, *Haemophilus*	↓ *Bacteroides*, *Eubacterium*, *Alistipes*	↑ *Haemophilus parainfluenzae*, *Streptococcus anginosus*, *V.atypica*, *V.dispas*, *Veillonella sp*. Oral taxon, *C.perfringens*	↓ *F.prausnitzii*, *Coprococcus comes*	[52]
Liver cirrhosis	Stool	16S rRNA multitag pyrosequencing (MTPS) + LH-PCR			↑ Lactobacillaceae, Enterococcaceae, Enterobacteriaceae	↓ Clostridiales XIV, Lachnospiraceae					[53]
Liver cirrhosis	Stool	16S rRNA multitag pyrosequencing (MTPA)			↑ Enterobacteriaceae	↓ Lachnospiraceae, Ruminococcaceae, Clostridiales XIV, Erysipelotrichaceae					[54]
Liver cirrhosis	Saliva	16S rRNA multitag pyrosequencing (MTPA)			↑ Furobacteriaceae, Prevotellaceae, Enterococcaceae, Enterobacteriaceae	↓ Lachnospiraceae, Ruminococcaceae, Clostridiales XIV, Erysipelotrichaceae					[54]
Liver cirrhosis	Stool	16S rRNA sequencing (Illumina MiSeq)							↑ unknown *Peptostreptococcaceae*	↓ Unknown *Clostridiales*, *Roseburia faecis*, *Alistipes putredinis*, unknown *Oscillospira*, unknown *Mogibacteriaceae*, unknown *Dehalobacterium*	[55]
Liver cirrhosis + Ascites	Stool	16S rRNA sequencing (Illumina MiSeq)				↓ Unknown Ruminococcaceae, Clostridiales, Peptostroptococcacea			↑ *Veillonela dispar*	↓ *R.faecis*, *A.putredinis*	[55]
Liver cirrhosis	Stool	16S rRNA multitag pyrosequencing (MTPS) and LH-PCR			↑ Enterobacteriaceae, Veillonellaceae	↓ Lachnospiraceae, Ruminococcaceae, Rikenellaceae		↓ *Blautia*			[56]
**Microbiota Composition in HCC Patients Compared with Healthy Controls**											
**Patients**	**Sample**	**Method**	**Family Level**		**General Level**				**Specie Level**		**Reference**
			**Increased**	**Decreased**	**Increased**		**Decreased**		**Increased**	**Decreased**	
NAFLD + Cirrhosis+ HCC	Stool	16S rRNA sequencing	↑ Bacteroidaceae, Streptococcacea, Enterococcaceae, Gemellaceae	↓ Verrucomicrobiaceae, Bifidobacteriaceae	↑ *Bacteroides*, *Phascolarctobacterium*, *Enterococcus*, *Streptococcus*, *Gemella*, *Bilophila*		↓ *Akkermansia*, *Bifidobacterium*, *Dialister*, *Collinsella*, *Adlercreutzia*				[37]
Non-Viral aetiology+ Cirrhosis + HCC	Stool	16S rRNA Sequencing (Illumina Truseq)			↑ *Escherichia*-*Shigella*, *Proteus*, *Lachnospiraceae* UCG 010, *Veillonella*, *Subdoligranulum*, *Prevotella 2*, *Barnesiella and Ruminococcaceae spp.*		↓ *Buchnera*, *Megamonas*, *Lachnospira*, *Eubacterumventriosum and Lachnospiraceae* UCG 001				[57]
Different aetiologies + Cirrhosis + HCC	Stool	Analysis by CFU							↑ *E.coli*		[58]
Different aetiologies + Cirrhosis + HCC	Stool	16S rRNA Sequencing (Illumina MiSeq)			↑ *Klebsiella*, *Haemophilus*		↓ *Alistipes*, *Phascolarctobacterium*, *Rumnocccus*				[59]
Hepatitis B + HCC	Stool	16S16S rRNA sequencing (Illumina HiSeq) rRNA			↑ *Bacteroides*, *Veillonella*, *Phenylobacterium*, *Synechococcus*						[42]

**Table 2:** Therapeutics in rodent experimental models and patients.

Effect of Antibiotics Treatment in Rodents				
Model	Disease	Treatment/Antibiotic	Effect	Reference
Wistar Rats exposed to Ethanol	Alcohol-induced liver injury	Polymixin B and Neomycin	Treatment with antibiotics in rats with alcohol-induced liver injury: ◦ reduced the endotoxin levels in plasma ◦ reduced aspartate aminotransferase levels ◦ reduced the hepatic pathological score ◦ prevented hypoxia	[88]
Sprague–Dawley rats exposed to ethanol	Alcohol-induced liver injury	Ampicillin and neomycin	Treatment with antibiotics in rats with alcohol-induced liver injury: ◦ inhibited the effect of ethanol ◦ reduced the endotoxin levels in plasma	[89]
C57Bl/6 Mice under a High Fat Diet (HFD)	NAFLD	Bacitracin, neomycin and streptomycin (BNS)	Treatment with antibiotics in mice with NAFLD: ◦ increased tauro-b-muricholic acid levels ◦ inhibited FXR signaling in the ileum ◦ reduced hepatic lipid accumulation	[90]
Wistar rats exposed to CCL4	Cirrhosis	Norfloxacin and Vancomycin	Treatment with antibiotics in mice with liver cirrhosis: ◦ reduced intestinal mucosa inflammation ◦ reduced gut bacterial translocation ◦ restored intestinal permeability	[91]
Sprague–Dawley Rats exposed to DEN	HCC	Polymyxin B and Neomycin	Treatment with antibiotics in rats with HCC: ◦ reduced the levels of LPS in plasma ◦ reduced TNF alpha and IL6 levels ◦ reduced liver fibrogenesis and HCC multiplicity ◦ reduced levels of cell proliferation in tumor	[83]
C57Bl/6 Mice exposed to DEN/CCL4	HCC	Ampicillin, Vancomycin, Neomycin sulfate and Metronidazole	Treatment with antibiotics in rats with HCC: ◦ reduced tumor number, size and liver/body weight ratio ◦ reduced expression of cell cycle, fibrosis and inflammatory genes ◦ increased liver injury	[81]
C57Bl/6 Mice under a HFD	Obesity-related HCC	Ampicilin, Neomycin, Metronidazole, vancomycin	Treatment with antibiotics in rats with obesity-related HCC: ◦ reduced HCC development ◦ reduced senescent hematopoietic stem cells	[82]
**Effect of Antibiotics Treatment in Patients**				
**Patients**	**Disease**	**Treatment/Antibiotic**	**Effect**	**Reference**
Patients with recurrent hepatic encephalopathy resulting from chronic liver disease	Cirrhosis and Hepatic Encephalopathy	Rifaximin	Treatment with antibiotics in patients with liver cirrhosis: ◦ reduced the risk of suffering hepatic encephalopathy ◦ reduced the risk of hospitalization associated with hepatic encephalopathy	[92]
Patients with alcohol-related decompensated cirrhosis and ascites	Cirrhosis	Rifaximin	Treatment with antibiotics in patients with liver cirrhosis: ◦ reduced the risk complications such as variceal bleeding, hepatic encephalopathy, spontaneous bacterial peritonitis and hepatorenal syndrome ◦ increased the five-year probability of survival	[93]
Patients with cirrhosis	Cirrhosis	Rifaximin	Treatment with antibiotics in patients with liver cirrhosis: ◦ increased beneficial bacteria *Klebsiella*	[94]
Patients with cirrhosis	Cirrhosis	Norfloxacin	Treatment with antibiotics in patients with liver cirrhosis: ◦ reduced the episodes complications such as spontaneous bacterial peritonitis and hepatorenal syndrome ◦ increased the one-year probability of survival	[95]
**Effect of Probiotic Administration in Rodents**				
**Model**	**Disease**	**Treatment/Probiotic**	**Effect**	**Reference**
C57Bl/6N Mice exposed to ethanol	Alcoholic Liver Disease (ALD)	*L.rhamnosus* GG (LGG)	Probiotic administration in mice with alcoholic liver disease: ◦ prevented microbiome changes during the disease ◦ restored tight junction protein expression ◦ reduced endotoxemia and hepatic injury	[96]
C57Bl/6N Mice exposed to ethanol	Alcoholic Liver Disease (ALD)	*L.rhamnosus* GG (LGG)	Probiotic administration in mice with alcoholic liver disease: ◦ reduced hepatic inflammation and liver injury ◦ reduced TNF alpha expression ◦ inhibited TL 4 and TLR5-mediated endotoxin activation	[97]
Sprague–Dawley rats under a HFD	NAFLD	*Lactobacillus paracasei* B21060	Probiotic administration in rats with NAFLD: ◦ reduced liver inflammation markers ◦ reduced cytokines synthesis ◦ reduced steatosis ◦ preserved gut barrier integrity ◦ reduced the relative amount of Enterobacterales and *E.coli* in colonic mucosa	[98]
C57BL/6J mice exposed to fructose	NAFLD	*L.casei* Shirota (Lcs)	Probiotic administration in mice with NAFLD: ◦ reduced steatosis ◦ reduced alanine-aminotransferase (ALT) levels ◦ reduced the activation of TL 4	[99]
C57BL6/N mice with subcutaneous tumor injection	HCC	*L.rhamnosus* GG (LGG), viable *E.coli* Nissle 1917 (EcN), and heat-inactivated VSL#3 (1:1:1)	Probiotic administration in mice with HCC: ◦ reduced tumor growth, size and weight ◦ reduced Th17 cells in the tumor ◦ reduced e-cadherin and growth factors (TGF-b) ◦ increased beneficial bacteria with anti-inflammatory properties ◦ increased IL-10 ◦ increased HIF-1 expression	[100]
Sprague–Dawley rats exposed to DEN	HCC	VSL#3	Probiotic administration in rats with HCC: ◦ inhibited the translocation of endotoxins and reduced intestinal inflammation ◦ reduced bacterial dysbiosis and maintained intestinal integrity ◦ decreased tumor growth and multiplicity	[84]
**Effect of Probiotic Administration in Patients**				
**Patients**	**Disease**	**Treatment/Probiotic**	**Effect**	**Reference**
Obese children	NAFLD	VSL#3	Probiotic administration in children with cirrhosis: ◦ reduced the severity of NAFLD ◦ decreased HOMA and ALT levels ◦ increased GLPp-1 and aGLP1	[101]
Patients with NAFLD or alcoholic liver disease	NAFLD or alcoholic liver cirrhosis	VSL#3	Probiotic administration in both patients with NAFLD or alcoholic liver disease: ◦ improved plasma levels of MDA and 4-HNE ◦ reduced levels of aspartate aminotransferase (AST) and ALT Probiotic administration in patients with alcoholic liver disease: ◦ improved cytokine levels (TNF alpha, IL-6 and IL-10)	[102]
Patients with alcoholic psychosis	Alcohol-induced liver injury	*B.bifidum* and *L.plantarum 8PA3*	Probiotic administration in patients with alcoholic liver injury: ◦ increased beneficial bacteria such as Bifidobacteria and *Lactobacilli* ◦ reduced levels of AST and ALT	[103]
Patients with alcohol cirrhosis	Cirrhosis	*L.casei* Shirota *(Lcs)*	Probiotic administration in patients with alcoholic cirrhosis: ◦ reduced sTNFR1, sTNRF2 and IL10 levels ◦ reduced TLR2, 4 and 9 expressions ◦ increased phagocytic capacity	[104]
Patients with cirrhosis and hepatic encephalopathy	Cirrhosis	VSL#3	Probiotic administration in patients with cirrhosis: ◦ reduced episodes of hepatic encephalopathy ◦ reduced hospitalization risk ◦ improved Child–Turcotte–Pugh and model for end-stage liver disease scores	[105]
Patients with cirrhosis	Cirrhosis	*E.coli Nissle*	Probiotic administration in patients with cirrhosis: ◦ increased beneficial bacteria such as *Lactobacillus* sp. and *Bifidobacterium* sp. ◦ decreased potential pathogenic bacteria ◦ reduced endotoxemia and bilirubin levels ◦ improved liver functions evaluated by Child–Pugh score	[106]
**Effect of FMT Administration in Rodents**				
**Model**	**Disease**	**Treatment/FMT**	**Effect**	**Reference**
Sprague–Dawley rats exposed to CCL4	Hepatic encephalopathy	FMT form healthy donor	FMT administration in rats with hepatic encephalopathy: ◦ prevented hepatic necrosis ◦ reduced intestinal mucosal barrier damage and intestinal permeability ◦ improved hepatic encephalopathy grades and behavior ◦ reduced TLR4 and TLR9 expression ◦ decreased IL-1b, IL-6 and TNF alpha levels	[107]
C57Bl/6 mice exposed to ethanol	Alcoholic liver disease	FMT from alcohol-resistant donor mice	FMT administration in mice with alcoholic liver disease: ◦ avoided bacterial dysbiosis ◦ restored gut homeostasis ◦ prevented steatosis and liver inflammation	[108]
C57Bl/6 mice under a HFD	NASH	FMT from healthy donor	FMT administration in mice with NASH: ◦ increased of beneficial bacteria Christensenellaceae and *Lactobacillus* ◦ improved tight junctions and endotoxemia ◦ reduced lipid accumulation, proinflammatory cytokines and NAS score	[109]
**Effect of FMT Administration in Patients**				
**Patients**	**Disease**	**Treatment/FMT**	**Effect**	**Reference**
Patients with metabolic syndrome	metabolic syndrome	FMT form healthy donor	FMT administration in patients with metabolic syndrome: ◦ increased insulin sensitivity ◦ increased butyrate-producing intestinal microbiota ◦ decreased fecal short fatty acids	[110]
Patients with cirrhosis and Hepatic encephalopathy	Cirrhosis and Hepatic encephalopathy	FMT from donor with the optimal microbiota deficient in Hepatic encephalopathy	FMT administration in patients with cirrhosis and hepatic encephalopathy: ◦ improved cognitive function ◦ increased beneficial bacteria Lactobacillaceae, Bifidobacteriaceae ◦ increased Ruminococcaceae	[111]
Patients with decompensated cirrhosis and hepatic encephalopathy	Cirrhosis and Hepatic encephalopathy	FMT from a donor enriched in Lachnospiraceae and Ruminicoccaceae	FMT administration in patients with cirrhosis and hepatic encephalopathy: ◦ restored antibiotic-associated disruption in microbial diversity and function ◦ increased abundance of Neisseriaceae and Pasteurellaceae ◦ reduced Bifidobacteriaceae, Lachnospiraceae and Ruminococcaceae	[112]
Patients with advanced cirrhosis	Cirrhosis	FMT from healthy donors	FMT administration in patients with advanced cirrhosis: ◦ was demonstrated to be safe ◦ reduced ammonia plasma levels	[113]
